# Nanostructured Ceria: Biomolecular Templates and (Bio)applications

**DOI:** 10.3390/nano11092259

**Published:** 2021-08-31

**Authors:** Petr Rozhin, Michele Melchionna, Paolo Fornasiero, Silvia Marchesan

**Affiliations:** 1 Chemical and Pharmaceutical Sciences Department, University of Trieste, 34127 Trieste, Italy; petr.rozhin@phd.units.it (P.R.); pfornasiero@units.it (P.F.); 2 Unit of Trieste, INSTM, 34127 Trieste, Italy; 3 Istituto di Chimica dei Composti Organometallici, Consiglio Nazionale delle Ricerche (ICCOM-CNR), 34127 Trieste, Italy

**Keywords:** ceria, nanoparticles, nanorods, nanosheets, nanozyme, biomolecule, template, catalysis, anti-oxidant, oxygen radicals

## Abstract

Ceria (CeO_2_) nanostructures are well-known in catalysis for energy and environmental preservation and remediation. Recently, they have also been gaining momentum for biological applications in virtue of their unique redox properties that make them antioxidant or pro-oxidant, depending on the experimental conditions and ceria nanomorphology. In particular, interest has grown in the use of biotemplates to exert control over ceria morphology and reactivity. However, only a handful of reports exist on the use of specific biomolecules to template ceria nucleation and growth into defined nanostructures. This review focusses on the latest advancements in the area of biomolecular templates for ceria nanostructures and existing opportunities for their (bio)applications.

## 1. Introduction

Ceria is among the most studied metal oxides and it has attracted researchers’ interest for its ability to capture, store, and release oxygen, and has been widely applied to ”clean-air” catalytic conversion technologies, and, more generally, in catalysis [1,2]. Nanosized ceria can mimic a variety of enzymatic activities, earning the name of “nanozyme”, and it can catalyze chemical reactions with potential biomedical applications, for instance for sensing or reducing oxidative stress in pathological conditions [3].

**Figure 1:**
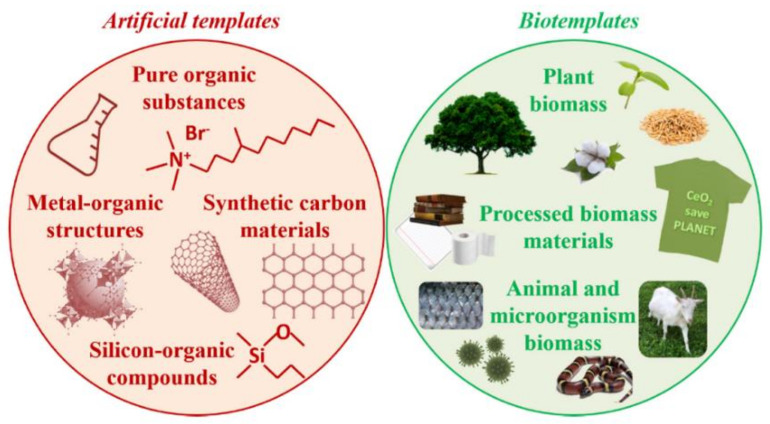
The search towards either artificial or bio-based templates to exert control over the growth of ceria has been very active. Reproduced from [4].

Ceria nanomorphology is an important factor affecting its reactivity, as described further below. It is thus not surprising that the search has been very active for suitable templates (Figure 1) to exert control over ceria nucleation and growth, and the topic of artificial or bio-based, hard or soft, templates for ceria has been recently reviewed [4]. In addition, microbial cultures of *Bacillus subtilis* were successfully used as bioreactors for the conversion of cerium (III) nitrate to ceria nanoparticles (NPs) [5]. The use of plant extracts for the green synthesis of nanoceria exploiting naturally occurring redox-active agents, such as polyphenols, is well-established [6]. Various plant parts have been used to this end, such as seeds [7,8,9], nut shells [10], leaves [11,12,13,14], flowers [15,16,17], bean sprouts [18], fruits [19,20], and kapok fibers (lignin) [21]. The use of anisotropic biotemplates, such as cotton, can be used to reproduce their morphology in ceria, e.g., microfibers [22], although the concept is typically applied at the microscale rather than nanoscale. Since green protocols for the preparation of nanosized ceria, along with its biological applications, were reviewed in 2017 [23], this work will focus on the latest developments in this area since then. It is worth noting that the vast majority of biotemplates, as discussed in the literature until now, have been obtained using top-down approaches, such as grinding or extraction, or through the use of microscale structures, usually consisting of mixtures of compounds. By contrast, the opportunities offered by pure biomolecules and their folding or self-assembly in bottom-up approaches have yet to be deeply explored, and to our knowledge, they have not been reviewed until now based on the molecular classes they belong to (i.e., carbohydrates, proteins, nucleic acids, etc.). This is particularly important also considering the catalytic activities of nanosized ceria that are responsible for the observed biological effects, and how they are affected upon binding to biomolecules. In this work, we gather the detailed knowledge pertaining to the interactions between specific biomolecules and ceria, both for the formation of nanoceria and for its (bio)catalytic activity. Furthermore, we discuss relevant examples described in the last five years to outline potential applications for these materials, with an emphasis on the biological ones to maximize the benefits offered by using biomolecular templates to attain nanosized ceria.

## 2. Ceria Nanomorphology-Reactivity Relationship 

The morphology of ceria nanostructures can exert an important influence on its properties and functions, and it is thus a key parameter to consider. Consequently, in the template-assisted synthetic protocol, the choice of the specific template is of great relevance. Ceria is typically formed from cerium (III) salts (such as nitrate or chloride precursors), and the nature of the anion was shown to be critical for the morphological control, for example leading to NPs or nanorods [24]. 

**Figure 2:**
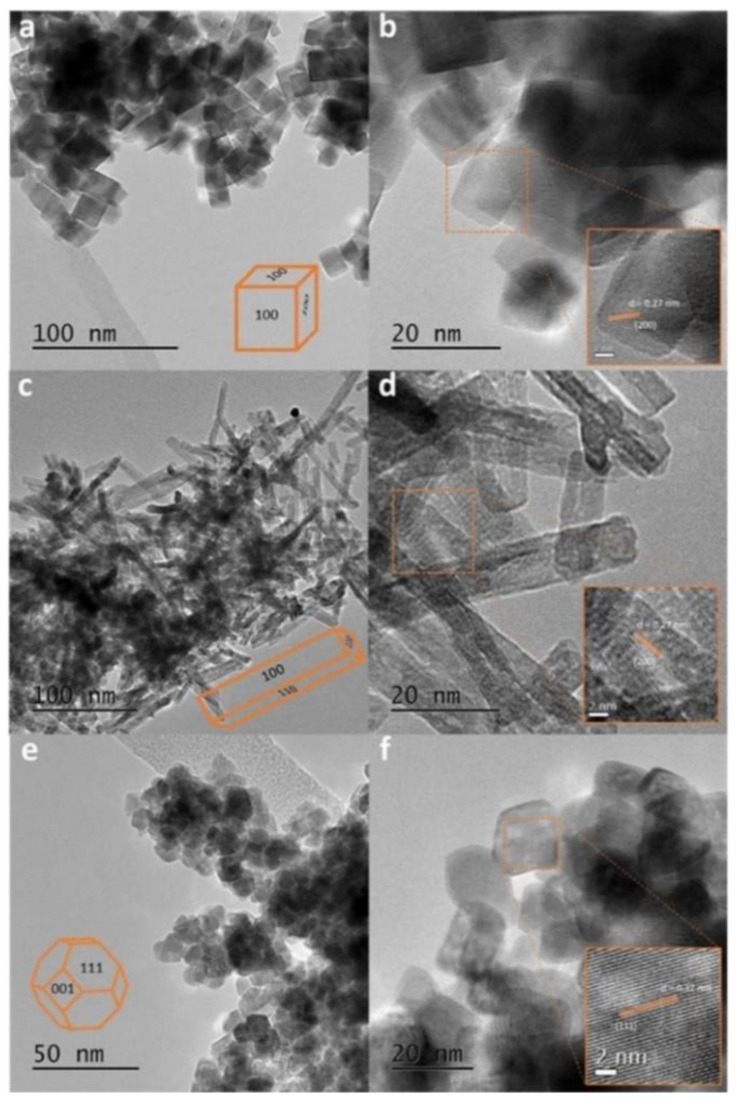
Transmission electron microscopy (TEM) images of ceria nanoparticles (NPs) with different morphologies: (**a**,**b**) cubic, (**c**,**d**) rodlike, and (**e**,**f**) polyhedral. Reproduced from [25].

A comparative study of cubic, rodlike, and polyhedral ceria geometries (Figure 2) on the catalytic conversion of glycerol into biorenewable methanol revealed that cubic particles displayed low catalytic activity. The low activity of the cubic NPs was ascribed to reduced surface area, relatively high acidity, and exclusive exposure of the (100) facet in the cubic geometry. This facet was more prone to hydroxylation under the reaction conditions, resulting in being detrimental for the investigated catalytic conversion [25]. Therefore, the nanocube morphology is a disadvantage for the catalytic production of methanol from glycerol. Different ceria nanomorphologies revealed varying levels of oxidase-like activity after interacting with DNA [26]. In particular, NPs and nanocubes demonstrated increased oxidase-mimetic activity upon binding DNA, whilst the opposite was true for nanorods [26]. Therefore, the former two geometries would be advantageous whenever DNA-binding could be a useful trigger to increase oxidase-like catalytic activity, for instance in biosensing. Conversely, a nanorod morphology would be disadvantageous for the same type of application. The structure–activity relationship of ceria nanorods, nanocubes, and nanooctahedra was studied for the generation of hydroxyl radicals through the catalytic decomposition of hydrogen peroxide. The reactivity was found to be highest for nanorods, followed by nanocubes and nanooctahedra. This trend was rationalized in terms of atomic defects, the percentage of surface Ce (III) ions, and the average coordination number of oxygen anions surrounding each cerium cation [27]. Therefore, nanorod or nanocube morphology could be advantageous to selectively trigger ROS-induced cell damage in pathological environments whereby there are higher levels of hydrogen peroxide, such as during inflammation.

Morphology maps revealed that the redox performance, particle size, and surface roughness could be optimized by engineering the oxygen vacancies’ density, which was influenced by the concentration of the precipitant/oxidant used during NP formation. These vacancies can be positioned at the surface, subsurface, or bulk regions, and it is the subsurface vacancies that are responsible for the main redox activity. These features can have important implications in the biological performance of nanosized ceria. For instance, ceria NPs exhibit antioxidant and cytoprotective effects at physiological pH 7.4. Conversely, at the acidic pH 6.4 that is typical of the tumor microenvironment, ceria NPs are oxidant and exert cytotoxic effects. The relative cytotoxicity thus depends on ceria nanomorphology with increasing levels in the order nanocubes < nanorods < truncated nanooctahedra [28]. This study suggested that in the case of osteosarcoma cells, truncated nanooctahedra was the ideal morphology to induce selective cytotoxicity. 

**Figure 3:**
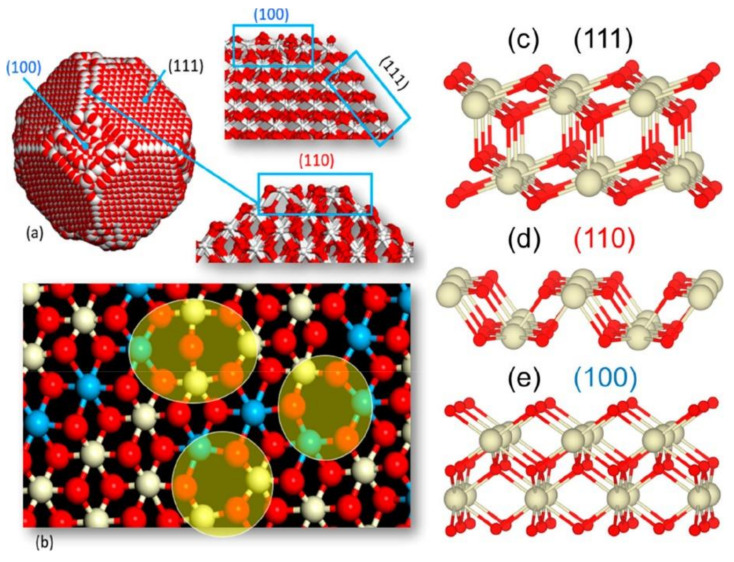
(**a**) Structure of ceria nanoparticle (NP) with (111), (110), and (100) surfaces. (**b**) View of one of the (111) surfaces on reduced nanoceria reveals oxygen vacancies (yellow ovals). The structures of defect-free (**c**) (111), (**d**) (110), and (**e**) (100) surfaces. Ce (IV) is white, Ce (III) is blue, and oxygen is red. Reprinted with permission from [29], Copyright © 2021, American Chemical Society.

A combination of experiments and in silico studies were used to design structures for nanoceria that maximize its catalytic activity. Polyhedral and nanocube morphologies expose active (100) surfaces (Figure 3), which should contain oxygen vacancies and surface hydroxyl groups. However, it was found that phosphate anions can strongly bind to (100) surfaces, inhibiting the oxygen capture and release, hence poisoning the ceria nanozyme. By contrast, the phosphate interaction with (111) surfaces is weaker, therefore these surfaces protect the ceria nanostructure against passivation [29].

## 3. Biomolecular Templates for Ceria Nanomaterials

Biotemplates have been attracting increasing interest to exert control over ceria morphology, although typically they are used for microscale (rather than nanoscale) assembly. They are often obtained through top-down approaches (e.g., grinding of biomass) and consist of mixtures of diverse molecules. A handful of studies instead focused on the use of specific biomolecules belonging to different chemical classes to template nanostructured ceria, as summarized in Table 1. These naturally derived molecules predominantly feature oxygen-bearing functional groups (e.g., carbohydrates, catechols, nucleic acids, proteins). Ligands on the surface of ceria NPs can have key effects on its antioxidant properties and even reverse it [30], therefore their choice should be carefully evaluated.

### 3.1. Carbohydrates

Considering the affinity of ceria for oxygen, the choice for a suitable template for ceria nucleation often falls on biomolecules that are rich in hydroxyl groups, such as carbohydrates and catechols. For instance, *Aloe vera* gel [49] and xantham gum [50] have been used as a source of polysaccharides to template nanosized ceria. Cellulose is a popular choice, although typically in the form of biomass or plant parts [21], as opposed to the purified polymer, despite the fact that the defined composition of the latter is more promising to attain finer control over homogeneously sized NPs. A recent report used microcrystalline cellulose in a sol-gel protocol to produce 8-nm sized ceria nanocrystals with a very narrow size distribution of the resulting NPs [32]. Similarly, starch templated 7–8 nm-sized ceria nanocrystals that, depending on experimental conditions, formed 7–13 nm-sized NPs [35]. In another study, alginate was used both as a precursor and a template, by providing a ceria-alginate gel, whose thermal decomposition produced spherical ceria NPs with a size < 5 nm and presence of functional groups, whose spectroscopic signatures were ascribed to carbonate and carboxylates [31]. Chitosan is another polysaccharide that was used as a template and capping agent for ceria NPs using a sol-gel method [33]. However, it is worth noting that when alginate and chitosan were used to coat ceria NPs, their stability was negatively affected, as they showed a tendency to agglomerate and sediment, and their antioxidant activity was altered [51].

### 3.2. Catechols

**Figure 4:**
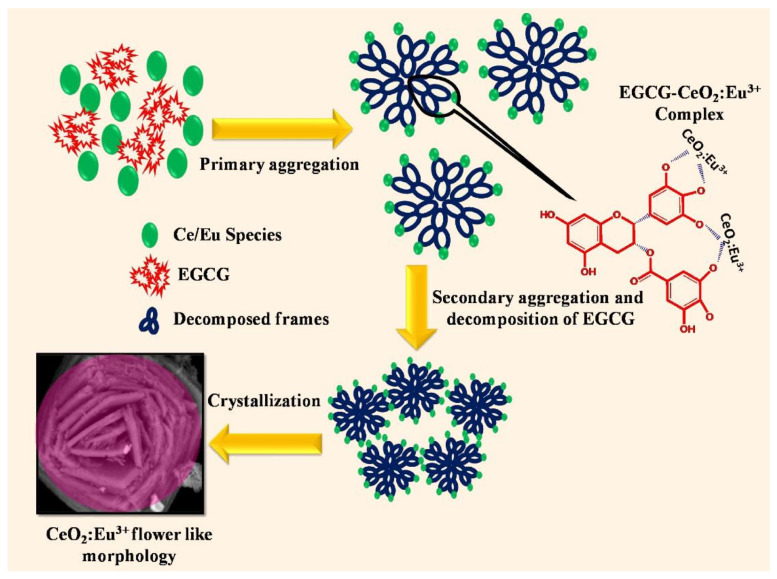
Epigallocatechin gallate (EGCG) biomolecular template for the nucleation and growth of ceria nanocrystallits that further assemble into a flower morphology. Reprinted from [38], Copyright © 2021, with permission from Elsevier.

Catechols are naturally occuring polyphenolic compounds that are widely known for their crosslinking and metal chelating abilities, as well as their redox chemistry [52,53]. One compound of this class that can be extracted from green tea and is attracting attention for its anti-oxidant properties is epigallocatechin gallate [54]. In a recent study, this template was successfully employed to form Eu-doped ceria flowers (Figure 4) composed of a few-nm-sized crystallites, and they have been applied for the luminescent detection of latent fingerprints [38]. 

Polydopamine (PDA) is another bioderived catechol that has gained widespread popularity in materials science, especially for its adhesiveness [55], which has proved effective in the generation of composites [56] and functional nanomaterials [57,58] with uses spanning from catalysis to theranostics [59]. The catechol groups of PDA NPs served to reduce first gold (III) to gold (0) onto the surface of the NPs, and then to anchor cerium (III) for the formation of ceria that found photocatalytic applications [36]. PDA was also used to coat reduced graphene oxide (rGO) and template ceria nanosheet formation [37]. 

### 3.3. Carboxylic Acids

Among carboxylic acids, the biocompatible and low-cost citric acid is possibly the most widely used capping agent and reductant for the preparation of metallic nanoparticles [60], and even beyond, to yield fluorescent biomaterials [61]. In the synthesis of nanosized ceria, the carboxylic moiety of citric acid can react with metal ions and form metallic citrate, with subsequent addition of ethanol leading to a gel. Interestingly, ceria crystallite size can be tailored depending on the calcination temperature used to form the metal oxide [39].

### 3.4. Phosphates and Nucleic Acids

**Figure 5:**
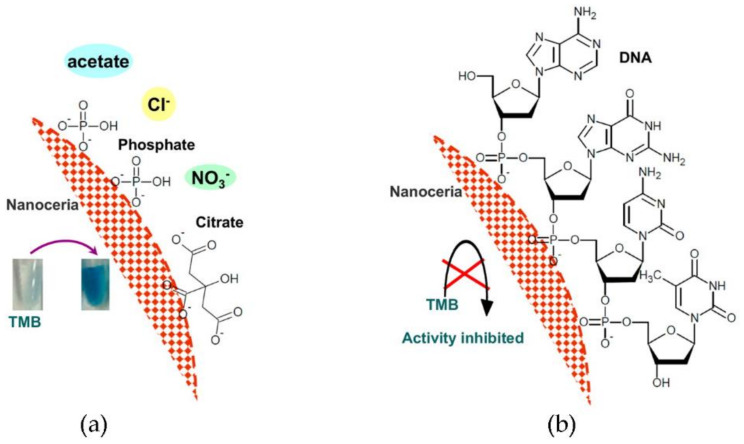
(**a**) Ceria binds with phosphate and citrate with higher affinity than acetate, chloride, or nitrate. In all these buffers, its oxidase-like activity is not affected, as it is able to convert 3,3′,5,5′-tetramethylbenzidine (TMB) into a blue product. (**b**) Ceria binds DNA through the phosphate groups independently from the DNA sequence and DNA adsorption impairs its oxidase-like activity. Reprinted with permission from [62], Copyright © 2021, American Chemical Society.

Ceria is well-known for its affinity to phosphate groups, as discussed further below in the relevant applications. Among the phosphate-containing biomolecules, nucleic acids are an obvious choice for the templating of nanoceria. DNA has been used as a biotemplate and capping agent for the nucleation of ceria nanocrystals since the major groove of the DNA double-helix was hypothesized to be appropriate both in size and chemical composition to nucleate ceria NPs. For instance, 5 nm-sized crystals with enhanced stability against agglomeration could be formed upon DNA-assisted CeO_2_ NP synthesis [40]. Indeed, it had been shown that nanoceria can adsorb the phosphate groups of DNA on its surface in a sequence-independent manner (Figure 5), although this interaction can lead to the inhibition of nanoceria oxidase-like activity [62]. However, the oxidase-like activity of nanoceria was shown to be enhanced to different extents in the presence of various nucleoside triphosphates (NTPs), with GTP exerting the highest effect, followed by ATP [63]. This effect was ascribed to the coupling of the oxidative reaction with NTP hydrolysis, catalyzed by the nanoceria phosphatase-like activity [63].

### 3.5. Proteins

Proteins have been far less used as templates for ceria NPs with respect to polysaccharides. Typically, proteins such as albumin are studied for their interactions with pre-formed ceria NPs, relevant to the formation of a biocorona that enhances NP colloidal stability [64,65] and can affect the mechanism of cellular uptake [66]. In particular, the adsorption of amino acids with carboxylic acid groups on their sidechains has been studied [67].

**Figure 6:**
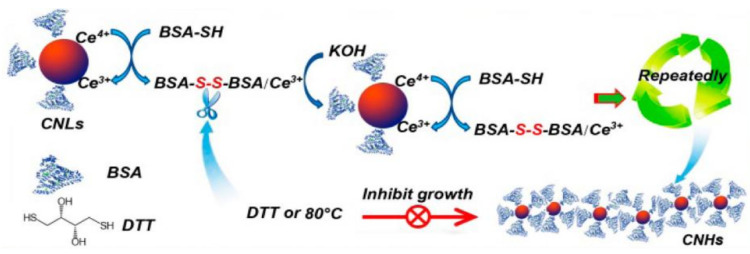
Proposed mechanism for the biomolecular templating effect of albumin on ceria NPs nucleation and growth into nanochains. Reprinted with permission from [45]. Copyright © 2021, American Chemical Society.

One work used albumin as a biomolecular template for the nucleation and growth of ceria spherical NPs, and their association into nanochains (Figure 6). Albumin displays disulfide bridges that could be reduced to thiols, thus promoting the concomitant oxidation of cerium (III) in the nitrate precursor to cerium (IV) oxide. The variation of experimental conditions, in particular the temperature used for the nucleation of the NPs, allowed them to fine-tune their final morphology, with cooling at 4 °C favoring 40 nm-long nanochains and heating at 80 °C promoting the formation of homogeneous 3.7 ± 0.7 nm spherical NPs [45].

In another study, bovine serum albumin was biomineralized with ceria to produce Ce-doped carbonaceous NPs that were envisaged for antioxidant therapy [44]. Glycine was also investigated as a very simple biotemplate to form ceria via a hydrothermal route. Varying the amino acid concentration led to differing morphologies of ceria microparticles, while the addition of ethanol as a co-solvent yielded homogeneously sized spherical NPs [68].

**Figure 7:**
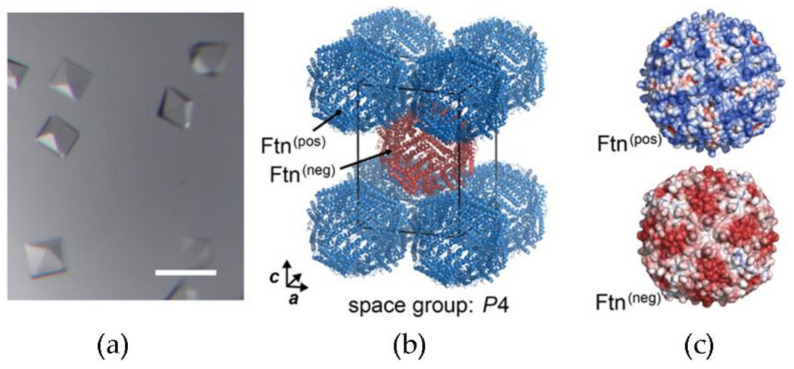
(**a**) Optical micrograph of crystals of engineered ferritin proteins to display a cationic (Ftn(pos)) or anionic (Ftn(neg)) surface, scale bar 200 μm. (**b**) Molecular structure of binary crystals. One unit cell of the tetragonal lattice is shown, protein backbone depicted in cartoon representation. (**c**) Electrostatic potential (red, −5 kT/e; blue, +5 kT/e) of Ftn(pos) and Ftn(neg), viewed along the 4-fold axis. Adapted with permission from [69]. Copyright © 2021, American Chemical Society.

Apoferritin is a ubiquitous protein for iron storage as ferric hydroxide NPs, with a distinctive container morphology with an inner cavity of 7 nm that was successfully used to nucleate ceria nanocrystals of 5 nm [46]. A superlattice was engineered from ferritin cages with inner cavities of 7 nm and charged surfaces (Figure 7) to nucleate the growth of ceria NPs, leading to oxidase-like and peroxidase-like catalytically active crystals [47,69].

Finally, silicateins are a class of proteins that has been widely applied for biomineralization, and although many researchers have attempted to identify the optimal peptide sequence for this purpose, usually the target is the preparation of silica NPs [70]. A remarkable, bioinspired approach exploited a mutated silicatein for optimal expression as a recombinant protein in *E. coli* was successfully used for templating the nucleation of ceria nanocrystals < 3 nm in size [48]. 

## 4. (Bio)applications

Nanosized ceria finds a large variety of applications [71], and extensive reviews exist on the topic, especially relevant to catalysis [1] for energy [72,73] and environmental preservation [74] and remediation [75,76]. Therefore, here we will focus on the latest advancements described in the most recent years, with an emphasis on biological applications, for which the use of biomolecular templates for ceria nanostructures’ assembly is particularly relevant.

### 4.1. Nanocarrier for Therapeutics

Nanomaterials are highly promising to innovate in medicine, thanks to their unique properties that arise from working at the nanoscale [77]. A fascinating opportunity is to use ceria NPs as nanovectors, as shown with a nanoemulsion obtained from lemon and corn oils that was envisaged for drug delivery [78]. Biomimetic lipids successfully yielded a vector for ceria NPs to cross the blood–brain barrier. Once internalized by neurons, they acted both as neuroprotective and pro-neurogenic agents, as demonstrated using co-culture systems [79]. NP size is a discriminating parameter for the cell uptake mechanism. NPs with a diameter as small as 3–5 nm can passively cross the membranes, a process relevant for the delivery of therapeutic biomolecules that could be otherwise negatively affected by the harsh chemical environments of endosomal pathways [80]. A fluorescent assay was developed to study ceria NP uptake by cells [81]. 

Ceria nanorods were shown to be able to deliver RNA interference (RNAi) to treat atherosclerosis, through silencing of the mTOR gene that controls autophagy and lipid metabolism. The use of a targeting peptide allowed for selective penetration into pathological plaques, PEGylation extended the nanocarrier’s circulation time, while the anisotropic morphology facilitated endosomal escape whilst ensuring the "on-demand" release of the RNAi cargo through competitive coordination of cytosolic hydrogen peroxide for gene therapy [82]. MicroRNAs are another type of therapeutic biologics that were envisaged to be delivered with ceria NPS as carriers to target directly the site of interest, such as the lung, and avoid systemic distribution [83]. 

### 4.2. Phosphoproteomics and Phosphatase-like Nanozymes

The area of phosphoproteomics is advancing at a rapid pace, in order to gain a better understanding of the biochemical profiling of several pathologies, especially for their early diagnosis [84]. However, phosphate groups are labile, making the detection of phosphopeptides quite a challenge, for which metal oxide affinity chromatography (MOAC) has offered promising solutions [85]. Amongst the various metal oxides, titania (TiO_2_) is widely applied, and the morphology of the nanocomposites has been revealed to be critical for the resulting MOAC performance [86]. However, ceria has been studied for the enrichment and detection of low-abundance phosphopeptides too [87,88]. A combination of its MOAC sorbent ability with its peroxidase-mimicking activity allowed the development of a colorimetric assay for phosphoproteins’ detection, since the nanozyme activity was reduced upon adsorption of the biomolecular target [89]. 

**Figure 8:**
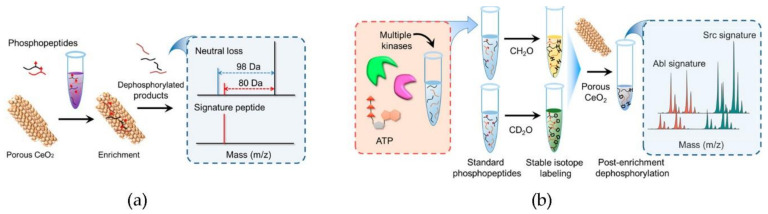
Multiplexed mass spectrometry approach for kinase biomarkers. (**a**) Ceria is used as MOAC sorbent and phosphorylase-mimic for phosphopeptide detection. (**b**) Ceria allows for the enhanced detection of Abl and Src kinases. Adapted with permission from [90]. Copyright © 2021, American Chemical Society.

A multiplexed quantitative matrix-assisted laser desorption/ionization mass spectrometry (MALDI MS) approach was developed to simultaneously assess the activity and inhibition of multiple protein kinases, which are an important class of cancer biomarkers (Figure 8). In particular, phosphorylated peptides that act as substrates for the kinases of interest were captured and dephosphorylated by ceria, allowing for enhanced detection. The method was successfully applied to Abl and Src, the kinases that are involved in chronic myeloid leukemia [90].

**Figure 9:**
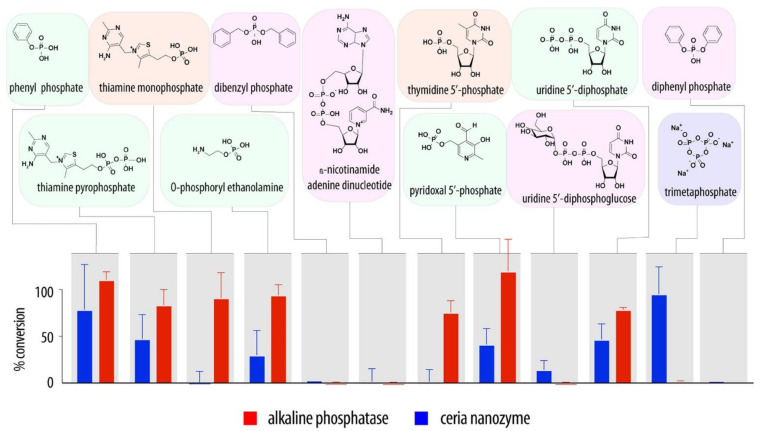
Substrate scope of ceria nanozyme, in comparison with alkaline phosphatase, for the hydrolysis of organic phosphates in a study for the delivery of phosphate prodrugs. Reproduced with permission from [91], Copyright © 2021, American Chemical Society.

Indeed, the ability of ceria to adsorb phosphate can be used to design phosphatase-mimics and applied to the conversion of phosphate prodrugs into active therapeutics (Figure 9), including chemotherapeutics for advanced cancer therapy [91]. It also proved to be efficient in the dephosphorylation of thiamine pyrophosphate first to thiamine monophosphate, and then to thiamine, thus producing the free vitamin form [92].

Besides, this nanozyme activity can be applied to the decomposition of pollutants, such as phosphate-bearing nerve agents [93,94]. Phosphatase-like activity is extensively studied to induce DNA cleavage, with potential applications in DNA repair, gene editing, and biosensing, as recently reviewed [95]. The interaction of polyphosphates bearing various phosphate units with nanoceria was studied ad it was shown that their esterification significantly reduces affinity for ceria [96]. Phosphate efficiently displaced DNA from ceria, contrarily to phosphite and hypophosphite. This observation could be used to screen for differing phosphorus species or oxidizing agents [97].

Ceria NP morphology was shown to affect its nanozyme activity, probed as phosphatase. In particular, ceria nanofibers were more active than commercial nanoceria and nanopolyhedra, while nanocubes displayed negligible activity. The results were correlated with the higher amount of Ce (IV) in the nanofibers that could bind hydroxide and phosphate to catalyze hydrolysis. This also meant that the catalytic activity was dependent on the buffer used, being completely quenched in phosphate buffer, and preserved in TRIS, glycine, or HEPES buffers. Furthermore, the nanozyme could adsorb enzymes or antibodies and was envisaged as a protein vehicle [98]. However, another study demonstrated that through the appropriate formulation of ceria NPs, it is possible to avoid phosphate-induced inhibition of catalytic activity [99].

Ceria NPs catalyzed the hydrolysis of 3′,5′-cyclic adenosine monophosphate (cAMP), which is a second messenger, involved in a plethora of signal transduction pathways. Importantly, the catalysis was only slightly affected by the pH, and highly specific to ceria, as opposed to other lanthanide oxides (i.e., La_2_O_3_, Pr_6_O_11_, and Nd_2_O_3_). The unusual phosphatase mimicry arose from an interplay between properly positioned Ce (III) and Ce(IV) cations, as well as cerium-activated hydroxyl moieties [100].

### 4.3. Photocatalysis and Catalysis

Ceria is a well-known material for photocatalytic processes, such as dye and drug photocatalytic degradation for environmental remediation [16,38,101]. To this end, ceria has been combined with ferrihydrites for the photo-Fenton degradation of tetracycline or other model pollutants (i.e., tetrabromobisphenol A, Rhodamine B, and 2,4-dichlorophenol) via the generation of reactive hydroxyl radicals [102]. Ceria NPs have been applied to the catalytic ozonation of phenol too, as a model pollutant [32].

**Figure 10:**
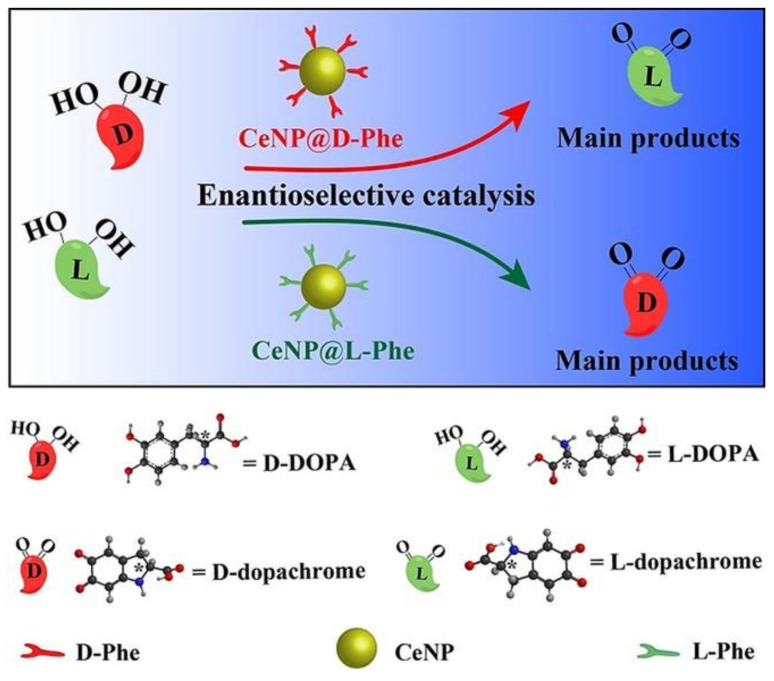
Stereoselective catalytic oxidation of DOPA by phenylalanine (Phe)-modified ceria NPs. The catalyst with D-Phe oxidized L-DOPA into L-dopachrome more effectively. Conversely, the catalyst with L-Phe oxidized D-DOPA into D-dopachrome more effectively. Reproduced with permission from [103], Copyright © 2021 Wiley-VCH Verlag GmbH & Co. KGaA, Weinheim.

Ceria NPs were coated with polyacrylic acid for the subsequent grafting of different amino acids. It was found that the use of Phe led to the best performance as chiral catalysts for the stereoselective oxidation of DOPA, a drug used in Parkinson’s disease, to dopachrome (Figure 10). Interestingly, while the use of enantiomers inverted the stereoselectivity as expected, it was found that the use of Phe as a chiral agent led to opposite stereoselectivity relative to His. Higher stereoselectivity was obtained for D-amino acids and it was rationalized in terms of their ability to engage in π–π interactions and H-bonding with the substrate [103].

Another interesting field of application is DNA repair. UV-induced damage includes the formation of cyclobutane pyrimidine dimers, which can be dissociated by photolyase enzymes, or nanosized ceria as their mimicry [104]. Protection from DNA damage was also reported through the ability of ceria to absorb ionizing radiation [105], combined with its antioxidant, as studied on irradiated cells [106].

Ceria NPs were used as supports for (2,2,6,6-tetramethylpiperidin-1-yl)oxy (TEMPO) free radical catalysts for the selective oxidation of primary alcohols of carbohydrates, and they demonstrated good stability and better performance than homogeneous catalysts over six consecutive runs [107].

The ability of nanosized ceria to promote the formation of radical oxygen species has been exploited in photocatalytic water oxidation, too [15,108]. For instance, a porphyrin photosensitizer was embedded in ceria nanotubes so that the electronic communication between the two components allowed for the enhanced production of molecular oxygen over the competing hydrogen peroxide generation from the partial water oxidation [109]. Nanosized ceria was also reported to enhance the performance of microbial fuel cells by creating oxygen reservoirs for the oxygen reduction reaction (ORR), while direct involvement of the cerium redox couple in the catalysis was only marginal [110].

### 4.4. Reactive Oxygen Species (ROS) Mitigation

#### 4.4.1. Mechanisms of Nanozyme Activity Pertaining to ROS Mitigation

Nanostructured ceria can mimic the activity of several enzymes, including superoxide dismutase (SOD) and catalase (CAT), which have been the subject of intense mechanistic investigations. SOD catalyzes the conversion of O_2_˙^−^ to H_2_O_2_, which then undergoes catalytic dismutation by the CAT into water and molecular oxygen. Interestingly, in the past, the SOD and CAT mimetic activity had been proposed to occur via direct electron transfer from O_2_˙^−^ or H_2_O_2_ to ceria with concomitant redox cycling between Ce (III) and Ce (IV) of surface sites by drawing an analogy with those of the natural enzyme. However, recent studies demonstrated that the redox potential of the Ce (III)/Ce (IV) couple is unfavorable for such a mechanism [111]. In the case of SOD-mimicking activity, the catalytic cycle requires surface defective sites and consists of two key steps, similarly to the SOD-mimicry by noble metals: (i) HO_2_˙ chemisorption onto the ceria surface and (ii) O_2_ and H_2_O_2_ generation. In the case of CAT mimicry, the mechanism is markedly different from that of noble metals, the latter occurring only with the aid of a pre-adsorbed OH group. In this case, the two key steps are (i) H_2_O_2_ oxidation by the CeO_2_ (111) surface, with the generation of O_2_ and the reduced H_2_-CeO_2_ (111) surface; and (ii) the subsequent reaction between another H_2_O_2_ molecule and H_2_-CeO_2_ (111), producing two water molecules [111]. 

Ceria nanomorphology also influences the nanozyme activity towards being CAT-like or SOD-like. In particular, (111)/(100) nanopolyhedra with a high concentration of Ce^4+^ ions promoted catalase mimicry. Conversely, (100) nano/submicron cubes and (111)/(100) nanorods that grew in the (110) longitudinal direction, both with high Ce(III) levels, enhanced SOD mimicry [112]. Interestingly, changing the temperature of preparation of Ce NPs allowed the modulation of their mimicking properties of multiple enzymes, namely superoxide dismutase (SOD), catalase (CAT), oxidase (OXD), peroxidase (POD), alkaline phosphatase (ALP) enzymes, as well as their ability to scavenge the 2,2-diphenyl-1-picrylhydrazyl (DPPH) free radical. In particular, the NPs with the highest level of antioxidant activity provided cells with cytoprotection against aging- and H_2_O_2_-induced oxidative damage; contrarily, those with pro-oxidant activity were proposed as candidates to induce cancer cell death [113]. The POD-like activity can be boosted by doping the CeO_2_ with several first-row transition metals (i.e., Mn, Fe, Co, Ni, and Cu), with maximal effect noted for Mn, followed by Co [114]. The POD mimicry by metal and metal oxide NPs has been recently reviewed [115]. The ceria precursor concentration represents an additional parameter to be carefully assessed, as it is a knob for controlling the particle size and the amount of Ce (III) sites at the surface, which also impact the type of nanozyme activity (oxidase-like or antioxidant) [116]. Atomic layer deposition (ALD) proved to be a promising approach to modulate the Ce (III)/Ce (IV) ratio through the adjustment of film thickness, analogously to what is observed for ceria NPs with differing diameter [117]. The combination of ceria with gold in core-shell NPs allowed for multi-enzyme mimicry that exhibited POD, CAT, and SOD activity that could be controlled through pH adjustment [118]. 

#### 4.4.2. ROS Mitigation for the Treatment of Cancer and Chemotherapy’s Consequences

**Figure 11:**
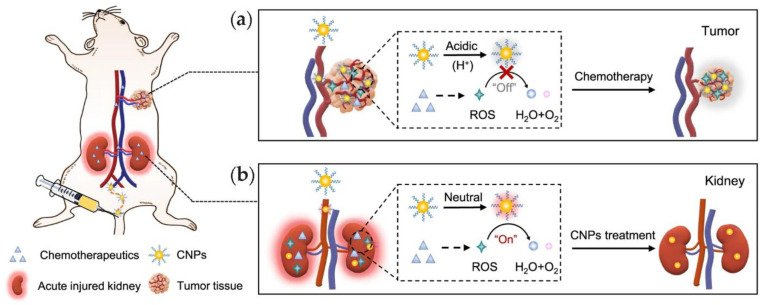
Schematic representation of pH-dependent redox activity of ceria NP as tested in vivo. (**a**) In the acidic tumor microenvironment, ceria NPs ROS scavenging ability is switched off and does not interfere with oxidizing chemotherapeutics; (**b**) in the neutral pH of the kidneys, ceria NPs scavenge ROS and exert cytoprotection. Reproduced from [119], under a Creative Commons license http://creativecommons.org/licenses/by/4.0/.

There are several pathological conditions that could be alleviated through ROS mitigation, and first and foremost, cancer is an obvious target. Furthermore, ROS can induce acute kidney injury in patients that receive chemotherapy. Ceria NPs can catalyze the dismutation of ·O_2_− into H_2_O_2_ at both neutral and acidic pH values, as well as decomposing H_2_O_2_ under neutral, but not acidic, pH. Therefore, ceria NPs exerted a general cytoprotection on cells that were challenged by oxidizing chemotherapeutics at a neutral pH, without compromising their anti-tumor activity at an acidic pH, which is a common feature of tumor microenvironments (Figure 11) [119]. Ceria NPs demonstrated a promising performance to partially revert the cellular mechanisms involved in tumor progression. They increased overall survival in vivo in rats with hepatocellular carcinoma, and following ex vivo uptake, perfused human livers and human hepatocytes. In vivo, ceria accumulated mainly in the liver, where it reduced inflammation and proliferation, and increased liver apoptotic activity [120]. 

Ceria NPs have also been inserted into the mesopores of dendritic silica-coated bismuth sulfide nanorods to impede their aggregation. The nanocomposite was proposed for the photothermal cancer therapy, through dual enzyme-activity mimicry. In particular, ceria was used to induce oxygen radicals-mediated cancer cell death, while the nanorods endowed the material with photothermal energy conversion ability using near-infrared light absorption of the bismuth sulfide nanorods. Furthermore, bismuth enabled high-contrast CT imaging [121]. Ceria NPs have also been proposed for the treatment of colon cancer, as it was noted that they can exert higher cytotoxicity in cancer cells relative to healthy cells through the generation of ROS and subsequent triggering of apoptosis [122]. In pancreatic cancer cells, ceria NPs acted as sensitizers to radiation therapy through activation of c–Jun kinase that promoted apoptosis, whilst protecting normal tissues from radiotherapy adverse effects [123].

#### 4.4.3. ROS Mitigation for Neurodegenerative Disorders

In Parkinson’s disease, ceria can selectively scavenge mitochondrial, intracellular, and extracellular ROS [124]. For Alzheimer’s, ceria has been combined with metal–organic framework (MOF) nanoparticles to attain multiple functions. They consisted of the co-delivery of small interfering RNA (siSOX9) and retinoic acid, as well as nanozyme activity mimicking superoxide dismutase (SOD) and catalase (CAT) enzymes based on the electron transfer between cerium (III) and cerium (IV). In vitro they promoted neural stem cells’ differentiation to neurons and alleviated oxidative stress, leading to reduced cell death and increased neurite length. In vivo they ameliorated cognitive impairment in an Alzheimer’s mouse model [125]. Finally, an artificial nanozyme, consisting of a ceria/polyoxometalate hybrid, displayed both proteolytic and SOD activities, so that it degraded amyloid β aggregates through oxidative damage and reduced intracellular ROS. Furthermore, it promoted PC12 cell proliferation, demonstrated an ability to cross the blood−brain barrier, and inhibited amyloid β-induced microglial cell activation. In addition, in vivo studies revealed a promising biocompatibility profile that paved the way to the development of multifunctional artificial nanozymes to treat neurological disorders [126].

#### 4.4.4. ROS Mitigation to Treat the Liver and the Kidneys

Ceria NPs’ ability to accumulate in the mononuclear phagocyte system (e.g., liver, spleen, and kidneys) can be used in an advantageous way for selective targeting of this nanosized therapy in areas of localized inflammation that require antioxidant treatment [127]. In particular, ceria NPs have been proposed to treat liver diseases thanks to their ROS and reactive nitrogen species (RNS) scavenging activity, as recently reviewed [128]. Another disease model where they have been studied is the non-alcohol-dependent, fatty liver, which is associated with impairment and inflammation of liver tissue, and which was alleviated in a rat model by ceria [129]. Treatment of hypoxia-induced acute kidney injury had been proposed for ceria combined with zirconia for enhanced radical scavenging activity [130].

#### 4.4.5. ROS Mitigation for Osteoporosis

Additionally, ROS have been implicated in osteoporosis, as they can induce osteoblast and osteocyte apoptosis, thus promoting osteoclastogenesis, which is the formation of bone-resorbing cells. The ability of ceria NPs to scavenge ROS translated into antioxidant and osteogenic properties that were thus proposed for the treatment of osteoporosis [131]. This type of activity could be combined with bone tissue regeneration as discussed further below.

#### 4.4.6. ROS Mitigation for Inflammatory Diseases and Immune System Regulation

Their ROS-scavenging activity was also proposed to treat rheumatoid arthritis [132], and to adjuvate in the treatment of traumas whereby blood transfusions are not an option, and nanocarriers to deliver hemoglobin are being studied as a possible alternative [133]. Ceria NPs have been stabilized with cyclodextrin that coated the NPs and encapsulated dithranol for the combined therapy of psoriasis, and they showed effective mitigation of ROS-induced damage in vitro and in vivo in a mouse model [34].

Cytotoxic CD8+ T cells (CTLs) play a key role to control intracellular pathogens as well as cancer. Their treatment with nanosized ceria led to higher production of cytokines, including interleukin-2 (IL-2) and tumor necrosis factor-a (TNF-a), higher release of effector molecules, higher killing activity, and stronger viral clearance capacity in vivo. Mechanistically, the treatment inhibited ROS production, and therefore promoted the activity of NF-KB signaling, overall promoting the cytotoxic activity of CTL cells [134].

ROS mitigation by ceria has been shown to limit the inflammatory response of overactivated microglia in a model of inflammation [135]. This effect could be beneficial to treat neuropathic pain, which is a chronic pathology that is caused by injury or dysfunction in the nervous system. An effective strategy to treat pain hypersensitivity employed microglia-targeting ceria-zirconia NPs that had been decorated with microglia-specific antibodies, enabling the rapid and effective inhibition of microglial activation, as demonstrated in a spinal nerve transection-induced neuropathic pain mouse model, proving the potent analgesic effect of the NPs [136]. Finally, lipid-coated magnetic silica NPs doped with ceria were designed for theranostics, as they allowed for MRI and mitigation of the inflammatory response of macrophages after being engulfed by these cells in a rodent model of intracerebral hemorrhage [137]. 

### 4.5. Reactive Nitrogen Species (RNS) Mitigation

Nitrogen stress can be caused by nitrogen excess or deficiency, and it is rather common in agriculture, where it can lead to impairment of plant growth. When tested in hydroponic rice stressed with altered nitrogen levels, ceria NPs mitigated the oxidative damage through the regulation of antioxidant enzymes, proline, and phytohormone levels. Interestingly, in the case of nitrogen deficiency, ceria NPs’ dissolution, and consequent uptake and accumulation by the plant, was increased, and it was ascribed to altered root biomass and increased presence of carboxyl compounds in the root exudates. However, at high concentrations, ceria NPs inhibited plant growth in the absence of stress in the hydroponic culture. Therefore, ceria NPs can exert positive effects but their concentration needs to be monitored carefully [138]. Furthermore, it has been postulated that nanosized ceria may interfere with plant–bacteria interactions at the root level, as demonstrated in a study on nitrogen-fixing symbiotic organisms [139].

### 4.6. Sensing 

#### 4.6.1. Ceria as Nanozymes for Sensing

Ceria NPs find many applications in sensors’ development, as recently reviewed [140]. In sensing, ceria NPs have been proposed as mimetics of various oxidizing enzymes, since they display increased stability relative to biological catalysts. To this end, they have been employed as glucose-oxidase mimics [141], but also peroxidase mimics in Enzyme-Linked Immunosorbent Assay (ELISA), as recently reviewed [142]. Ceria-titania mesoporous nanosheets’ SOD mimicry has been used also for the electrochemical detection of ·O_2_− for biosensing [37]. 

Ceria’s ability to mimic peroxidase, catalase, and oxidase enzymes [143] allows for the detection of hydrogen peroxide [144,145,146,147]. Hydrogen peroxide is produced by cancer cells and can be detected by ceria NPs in combination with CuCo_2_O_4_ nanosheets and carbon nanotubes for increased sensitivity [148]. Hydrogen peroxide is also produced by activated macrophages in atherosclerotic plaques, for which a ceria nanowire sensor was developed displaying fluorescent DNA for the competitive binding with hydrogen peroxide, as well as folic acid and CD36 antibody for cell targeting and imaging in vivo [149].

#### 4.6.2. Sensing for Drinking Water and Food Safety

Ceria NPs have been envisaged for the quality assessment of both drinking water and food. In the first type of application, ceria NPs have been used to develop an electrochemical sensor for arsenic (V) in polluted waters using DNA. Ceria coordinates the phosphate of the nucleic acid, albeit with lower affinity relative to arsenic (V), so the latter can displace the DNA from ceria NPs and generate an electrochemical signal [150,151]. The ability of ceria NPs to bind free DNA was also exploited to adsorb antibiotic-resistance genes from tap water to target this issue of public health concern [152].

**Figure 12:**
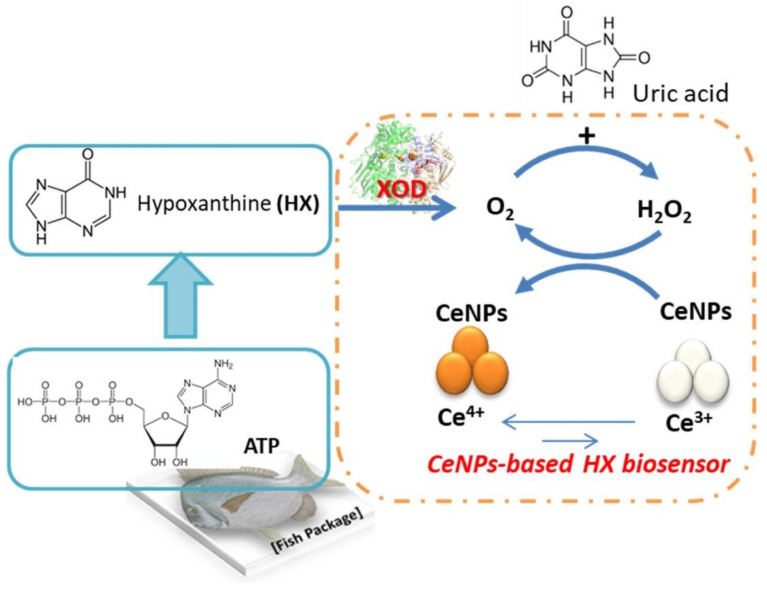
Ceria NPs (CeNPs) and xanthine oxidase (XOD) for the biosensing of hypoxanthine (HX) to monitor fish freshness. Reprinted from [153], Copyright © 2021, with permission from Elsevier.

With regards to the molecular target for detection, ceria NPs have been proposed to sense hypoxanthin, which is a product of nucleotide degradation in fish and meat that can be used to monitor food freshness [153]. In this case, the NPs were immobilized with xanthine oxidase onto the surface of silanized paper, which was placed in contact with fish extract, so that hypoxanthine could be converted into xanthine first, and then uric acid, with concomitant generation of hydrogen peroxide to form molecular oxygen. The peroxide could then be reduced by ceria NPs that in turn get oxidized to colored Ce (IV) in a colorimetric paper-based sensor for monitoring food quality (Figure 12) [153]. 

Cerium oxide nanorods were used to develop an electrochemical DNA biosensor to detect *Salmonella* for food safety applications [154,155]. In addition, DNA from *E. coli* could be detected using a ceria-based biosensor that exploited its oxidase-mimicking activity [156]. Ceria was also used in combination with graphene quantum dots to develop a sensor to detect ochratoxin A, with relevance to food safety [157]. 

#### 4.6.3. Sensing for the Detection of Disease Biomarkers

Ceria hollow NPs have also been proposed for sensing useful biomolecules for health monitoring. These not only included sensors for glucose [21,146,158,159,160], whose levels require careful monitoring for diabetic patients, but also the C-reactive protein [161], which is a biomarker for acute inflammation. Other sensing targets were the amyloid beta protein [162,163] and dopamine [164,165], both of which are relevant to neurodegenerative processes, and, in particular, Alzheimer’s and Parkinson’s disease, respectively. Ceria NPs were used to develop sensors for lactate, whose levels can be diagnostic of various diseases [166]. ATP [167], microRNAs [168,169], and DNA [170] were successfully detected too using ceria, for cancer diagnostics [171] or forensics applications [172]. Sensors were developed to monitor the presence of drugs, such as sulfonamide [173] and omeprazole [174]. Additional targets were a hypertension biomarker consisting of the epithelial sodium channel [175], and TNF-alpha for the early screening of neonatal diseases [176]. Ceria NPs were combined with iron oxide for the binding of carboxylic acid groups of a specific antibody that recognizes the carbohydrate antigen 19-9 as a cancer marker. In this case, the NPs were embedded in a mesoporous carbon matrix to develop an electrochemical sensor based on impedance variations for cancer detection [177]. 

#### 4.6.4. DNA Sensors

Ceria NPs’ affinity for phosphate groups and nucleic acids has been exploited in numerous DNA sensors. In one example, ceria NPs were anchored on gold nanorods that were combined with quantum dot-derivatized zinc oxide nanoflowers. The system was deposited on a paper working electrode to provide a photoelectric layer that was used to label one end of assistant DNA, for its subsequent hybridization with a capture probe to yield a triple helix. Incubation with a mixture of 4-chloro-1-naphthol and hydrogen peroxide resulted in peroxidase mimicry, leading to precipitation of the reaction product on the electrode and quenching of the signal. Conversely, the presence of the DNA triple helix led to the release of the gold nanorods-ceria NP system, thus removing their quenching effect, and leading to a photocurrent signal. Consequent migration of the nanozyme onto another area allowed for the conversion of a chromogenic substrate into a colored product for colorimetric visual detection [178]. 

In another example, ceria nanorods were coated with iridium nanorods because of their propensity to adsorb oxygen species, which could be beneficial for the peroxidase-like activity of ceria NPs. The nanomaterials were combined with an aptamer that recognizes a protein overexpressed on the surface of cancer cells for their detection. Sensitivity of the system was enhanced through the inclusion of DNA walker technology. DNA walker strands display RNAse-like activity and cleave the RNA of chimeric DNA/RNA oligonucleotides (D-RNA). The name comes from the fact that the strand displacement reaction causes the DNA walker strands to “walk forward” along the adjacent D-RNA, thus continuously cleaving it and amplifying the signal through the release of many probes [179].

Ceria NPs also display glucose-oxidase biomimicking ability, as they catalyze the oxidation of glucose to gluconic acid, with an activity that is more pronounced for smaller NPs with higher surface area. This property was exploited for the development of a sensor that could detect DNA products amplified through PCR. The amplified nucleic acids bound to ceria and led to NP aggregation, thus impeding the nanozyme activity. Therefore, in the presence of glucose, its levels could be related to the amplified DNA [141]. In another example, CuMn-CeO_2_ functionalized with luminol was successfully applied to develop an electrochemiluminescent biosensor to specifically detect Group B *Streptococci* from clinical vaginal and anal swabs [180].

### 4.7. Medical Implants

**Figure 13:**
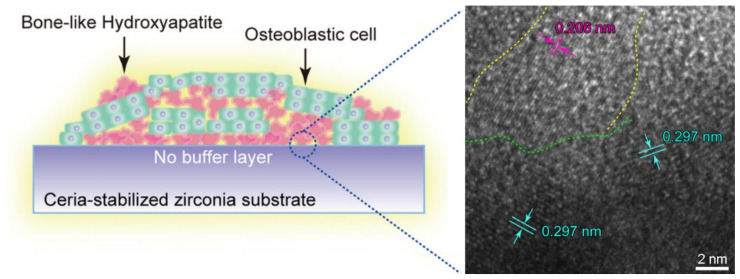
(**a**) Schematic representation of osteoblasti-cell (green) deposited hydroxyapatite (pink) into a ceria-zirconia substrate. (**b**) HR-TEM micrograph of the interface (green-dotted line) between the hydroxyapatite nanofibers (d = 0.206 nm of (400) face, magenta) and the zirconia nanocrystal (d = 0.297 nm of (101) face, cyan) at the nanoscale. Reproduced from [183], Copyright © 2021, with permission from Elsevier.

Ceria has been studied as an additive for well-known materials used in medical implants in virtue of its antioxidant and anti-inflammatory properties and to favor the direct osseointegration at the biointerface. This concept was applied to titania alloys [181], and nanotubes, and an increased level of early hydroxyapatite precipitation was noted from simulated body fluid [182]. Furthermore, ceria-stabilized zirconia has been proposed as a promising biomedical implant material. In particular, TEM imaging revealed zirconia nanocrystals directly bound to osteoblastic cell-precipitated hydroxyapatite crystals at the lattice-fringe scale, without any pretreatment of the substrate surface [183]. These results suggested the possibility for nanoscale direct osseointegration with bone in vivo (Figure 13) to improve the outcome of dental implants, as opposed to titanium surfaces that typically get coated by cell-secreted proteins [183]. 

The valence state of cerium is an important factor for the performance of ceria coatings on medical implants. Plasma spraying allows one to fine-tune the Ce (III)/Ce (IV) ratio in the resulting materials as shown on titanium devices. Interestingly, macrophages’ adhesion was restricted by a higher level of Ce (III) and promoted by an abundance of Ce (IV), which also resulted in an M2-type response associated with healing, as opposed to the M1 pro-inflammatory response, which was ascribed to the catalase-like activity of ceria that mitigates ROS [184]. 

### 4.8. Antimicrobial Activity

Ceria NPs exerted antimicrobial activity that was proposed for wastewater treatment [185], but also for wound-healing applications [14]. It can control biofilm formation and quorum sensing [186]. The antibacterial activity exerted by nanoceria was shown to be more effective on Gram-negative than Gram-positive strains [17]. Ceria demonstrated the ability to mimic haloperoxidase, and this property was applied to develop antimicrobials. In particular, a bioinspired strategy was used, since marine organisms rely on biohalogenation as a defense strategy against bacterial colonization, since it interferes with bacterial communication. Bismuth substitution enhanced the enzyme-like activity of ceria NPs three-fold, and the incorporation of the nanosystem into polyethersulfone beads, which are typical constituents of water filter membrane supports, led to decreased adhesion of the Gram-negative soil bacterium *Pseudomonas aeruginosa* and of *Phaeobacter gallaeciensis*, a primary bacterial colonizer in marine biofilms [187].

*Streptococcus mutans* forms biofilms that contribute to dental caries in the presence of fermentable carbohydrates and constitutes a target for therapeutic intervention. Ceria NPs reduced bacterial adhesion by 40%, and planktonic growth and dispersal assays supported a non-bactericidal mode of biofilm inhibition [188]. A detailed study of the bio–nano interface demonstrated that rod-like ceria NPs could be reduced by *Bacillus subtilis* under planktonic conditions, so that the Ce (III) ions adjacent to the surface oxygen vacancies would be chelated by the adsorption sites present on the bacterial cell wall. The bacterial biosorption of the dissolved Ce (III) ions unveiled a new mechanism for the toxicity of ceria NPs [189]. Ceria was also combined with zinc oxide onto halloysite nanotubes to reduce NP agglomeration and improve interfacial reactions between the nanocomposite and bacterial cells. The synergistic effects of the different components led to superior antibacterial activity of the nanocomposite against *Escherichia coli* [190].

Finally, the nitric oxide (NO) donor, *S*-nitroso-*N*-acetylpenicillamine (SNAP), was administered together with ceria NPs to induce synergistic antimicrobial effects against *Staphylococcus aureus*, *Escherichia coli*, and *Candida albicans*, as model organisms for Gram-positive bacteria, Gram-negative bacteria, and fungi, respectively. The best results were obtained with equimolar solutions of 3 mM for each agent and provided a promising outlook for the future development of broad-spectrum antimicrobials [191].

### 4.9. Tissue Engineering

The use of nanosized ceria for tissue engineering has been recently reviewed [192]. In particular, the ROS mitigation ability and positive interaction with hydroxyapatite nucleation render nanoceria highly promising for the regeneration of bone tissue. To this end, it was combined with an alginate-gelatin scaffold that demonstrated enhanced cell attachment and proliferation as well as the promotion of mesenchymal stem cell osteogenic differentiation [193], which was also shown for ceria NPs alone [194]. Pulsed laser deposition was applied to produce pyramid-shaped nanosized ceria for bone tissue regeneration. Modulation of the NP nuclei density allowed for the fine-tuning of the hydrophilic character of the material. The nanosized ceria induced a reorganization of the cell cytoskeleton in both osteosarcoma and osteoprogenitor cells, with the former showing elongated cell bodies and the latter increased adhesion, which might be beneficial for osteogenic differentiation [195].

Ceria NPs have been used to attain nanocomposites with hydroxyapathite for bone tissue regeneration [12]. Ceria NPs accelerated the formation of new bone and boosted endochondral ossification-based bone regeneration in both a subcutaneous ectopic osteogenesis model and a mouse model of critically sized bone defects. In particular, ceria NPs significantly promoted endochondral ossification-based bone regeneration by ensuring sufficient hypertrophic differentiation [196].

Ceria NPs have been applied to dental pulp regeneration. They were embedded as an insoluble antioxidant in a mineral trioxide aggregate that served as a biomaterial scaffold. Ceria accelerated odontoblastic differentiation of dental pulp stem cells via ROS downregulation, with minimal influence on the physico-mechanical properties of the scaffold [197]. ROS mitigation by nanoceria was also used for wound healing using a miRNA-loaded hydrogel to promote angiogenesis in the oxidative environment of diabetic wounds [198].

### 4.10. Energy Applications

Microalgae are an attractive source of biomass, and, potentially, biofuels. In particular, hydrothermal liquefaction is one of the main processes used to degrade lignocellulosic biomass into a crude bio-oil in hot compressed water. To this end, ceria NPs with a crystallite size of 6 nm were successfully applied as catalysts for the production of bio-oil from Spirulina platensis, in an attempt to exploit ceria’s ability to bind to oxygen-bearing organic compounds and catalyze their decomposition into biofuel [199]. Another study demonstrated efficient hydrothermal liquefaction of Scenedesmus obliquus microalgae into biofuel by using ceria nanorods decorated with nickel and activated carbon [200]. The same type of process was also catalyzed by ceria using rice straw as a starting material to produce bio-oil [201]. Ceria has been applied also as a catalyst for the pyrolysis of cellulose, which is the major constituent of lignocellulosic biomass, for the production of high-value bio-oil [202]. Carbon monoxide oxidation is another key reaction catalyzed by ceria nanocrystals [48], as well as carbon dioxide conversion into dimethyl carbonate [203].

### 4.11. Optoelectronics and Bioimaging

With human progress, we have witnessed an exponential increase in optoelectronic devices that require illumination. To this end, light-emitting diodes (LEDs) are highly attractive for their low-energy consumption, extended working lifetime, high efficacy, and environmental friendliness. In particular, lanthanides are promising to generate phosphors with chromatic tunability. Therefore, praseodymium was used to dope ceria nanopowders generated using *Aloe vera* as a fuel for a green combustion process, and yielded an orange-red light emitter [49]. Europium has been combined with ceria too, to yield red phosphors for optoelectronics [38]. 

Ytterbium and thulium were used to dope ceria NPs to exploit their near-infrared light absorptivity and up-conversion ability for deep-tissue imaging. Lanthanides are highly attractive for biomedical imaging thanks to their low toxicity, high stability, and minimized background auto-fluorescence. Furthermore, during the up-conversion, part of the absorbed light is converted into heat, thus upconverting NPs can serve as photothermal agents, potentially useful for concomitant sterilization during biomedical imaging. However, in vitro studies on *E*. *coli* as a bacterial model demonstrated a modest 44% sterilization efficacy when the doped NPs were irradiated at 980 nm [204]. Finally, ceria NPs have been embedded into electrospun chitosan nanofibers to endow them with fluorescence at 520 nm upon excitation at 430 nm for bioimaging [205].

### 4.12. Amyloidosis Inhibition

Amyloid-associated disorders constitute a large and diverse class of pathological states of growing societal concern, as the increasing life expectancy is leading to an increasing aged population, and consequent occurrence of amyloidoses. Amongst the many potential treatments, NPs have attracted growing interest as inhibitors of amyloid aggregation, including those formed by peptides [206], carbon nanostructures [207], gold [208], and also ceria. In particular, ceria NPs were shown to bind to the α-synuclein monomer and extend the lag phase time of amyloid fibril formation, and the resulting aggregates are relatively less toxic than those formed in the absence of ceria [209]. It will be interesting in the future to assess whether interesting new properties can arise from the use of other nanomorphologies, since it was shown that neurons’ interaction is favored when in contact on anisotropic, elongated nanomaterials (e.g., tubes, rods, fibers) [210]. 

**Figure 14:**
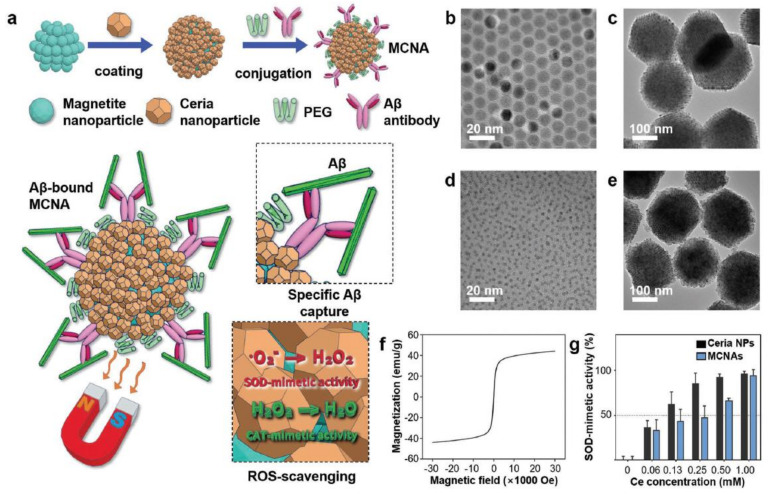
Magnetite/ceria NP assemblies (MCNAs). (**a**) Schematic preparation of MCNAs. (**b**–**e**) TEM images of magnetite NPs (**b**), their assemblies (**c**), ceria NPs (**d**), and MCNAs (**e**). (**f**) Magnetization curve of MCNAs. (**g**) SOD-mimetic activity of MCNAs. Reproduced with permission from [211], Copyright © 2021 WILEY-VCH Verlag GmbH & Co. KGaA, Weinheim.

Using a different approach, magnetite-nanoparticle assemblies were coated by nanosized ceria and further functionalized with anti-Aβ antibodies for body cleansing from amyloids. In this case, extracorporeal treatment of blood allowed for the selective capture of Aβ into the NP assemblies (Figure 14), which were separated form blood by using magnetic fields, and ceria allowed for the reduction of the oxidative stress generated by the concomitant immune response to the treatment [211]. 

### 4.13. Toxicological Studies

The widespread use of ceria in catalytic filters by the automobile industry has raised some concerns over potential undesired effects arising from its release in the environment. Therefore, the vast majority of toxicological studies have been focused either on aquatic or agriculture-relevant organisms. In the first group, studies have analyzed toxicity on diatoms [212], algae [213], clams [214], mussels [215], oysters [216], and zebrafish [217]. For instance, ceria NPs have been coated with polysaccharides (levan and pullulan) or a monosaccharide (glucose) and their toxicity was evaluated on three aquatic organisms: The bacterium *Vibrio fischeri*, the crustacean *Daphnia magna*, and zebrafish *Danio rerio*. The last one is a common model for toxicological studies on embryo development and revealed no adverse effects for either coated or uncoated NPs. The coating reduced the toxicity on bacteria and crustaceans, although it increased the bioaccumulation in the latter organism, for which respiration levels also appeared altered. However, no adverse effects were noted for NP concentrations up to 200 mg per liter [218]. Ceria NPs were also studied for their effects on river biofilm communities and were showed to exert selection pressure that ultimately altered the composition of the microbial community [219]. 

In the second group, nanosized ceria’s toxicity has been evaluated on agricultural crops [220], the tomato plant [221], hydroponic cucumber plants [222], and so on. Ceria NPs and released Ce (III) ions were also evaluated for their different mechanisms of inducing toxicity, in a study on plant growth and physiological and nutritional parameters relevant to applications in agriculture [223]. Ceria nanostructures demonstrated better biocompatibility towards plant tissues than analogous silver nanostructures [224]; however, transcriptome changes were noted in plants after exposure to nanoceria [225,226]. Ceria NPs’ effects were also noted on soil bacterial community composition [227] and rhizosphere biocommunities and were studied for their accumulation in soybeans [228]. An interesting study on ceria NPs applied to the leaves of common bean plants to evaluate adverse effects showed that both plant growth and development were not negatively affected, and no variation in the nutritional quality of the pods was noted other than mineral contents. However, dose-dependent oxidative damage occurred in the leaves, giving scope for further investigations on the biochemical effects of plants’ exposure to ceria NPs [229].

Positive effects of ceria NPs were instead noticed on tomato plant growth and metabolism up to 100 mg/L concentrations. However, higher levels (>200 mg/L) were detrimental to the growth and metabolism of the test plant and severe oxidative stress, with significant reduction in pigment, increased lipid peroxidation, electrolyte leakage, and H_2_O_2_ content. The activities of antioxidant enzymes were significantly upregulated [13]. This result may be attributed to SOD mimetic activity of nanoceria while the toxicity above the optimum concentration was probably attributed to the biotransformation of NPs and the high sensitivity of test seedlings exposed to the released Ce^3+^ ions. Further research is therefore required to gain deeper insights in understanding the uptake, accumulation, translocation, biotransformation, and toxicity of nanoceria in food crops, as well as the subsequent impact of possible transmission up the food chain.

Given the well-established use of ceria in car exhausts for the abatement of toxic gases, studies assessed the possibility of using ceria as diesel additives to reduce the emission of toxic compounds (such as benzo(a)pyrene) from the incomplete combustion of fossil fuels, and to increase fuel economy. As mentioned above, the potential release of the NPs in the environment required an accurate evaluation of the toxicity of nano-sized ceria, either alone or in combination with benzo(a)pyrene on reproductive cells revealing DNA damage [230]. This result was confirmed on sperm cells, where DNA damage [231] and oxidative stress due to ROS generation was found [232]. A report by Sundararajan et al. highlighted that *Drosophila* insects, in various developmental stages, were not liable to any significant toxicity in third instar larvae and adult flies over one month of continuous oral administration of ceria NPs up to 1 mM doses [233]. 

A route of potential exposure to nanosized ceria for humans is inhalation, therefore this type of exposure and potential pulmonary toxicity has been the subject of many investigations, in various models. Ceria NPs of heterogeneous size distribution ranging from a few nm to over 100 nm in diameter demonstrated no cytotoxicity on cell cultures exposed to NP aerosols [234]. The pulmonary and systemic effects of nanoceria exposure in mice were studied through repeated transnasal instillation, in order to assess the potential health risks posed by airborne nanoceria. In this study, 7-nm and 25-nm ceria NPs were used as representative models, for the most common ceria-NP fuel additives. Lung damage was manifest, with consequent penetration of nanoceria through the air–blood barrier, leading to NP distribution to other organs, especially the liver and the spleen. Interestingly, nanoceria could also reach the central nervous system through the olfactory nerve. Overall, the systemic accumulation triggered lipid peroxidation in multiple organs, with the smaller NPs inducing more severe pulmonary damage, albeit similar systemic toxicity, relative to larger ones [235]. Pulmonary exposure to ceria NPs aggravates vascular toxicity in rats with vascular injury induced by the anticancer cisplatin, through mechanisms that involve oxidative stress, inflammation, and DNA damage [236]. Inhaled ceria NPs can penetrate deeply into the lungs and it was shown that they can inhibit the formation of the pulmonary surfactant lining of alveoli. The effect depends on various physicochemical properties of the NPs, such as size, hydrophobicity, dissolution rate, and aggregation state at the biological interface [237]. However, no genotoxicity was noted in rat blood cells after inhalation of ceria NPs over 6 months [238].

Another potential route for ceria uptake is through the skin. Surprisingly there are scarce studies on this area. One recent work analyzed cerium oxide NPs dispersed in synthetic sweat using excised human skin on Franz cells and revealed very low dermal absorption and transdermal permeation of cerium [239].

Hemocompatibility and anticoagulant, anti-inflammatory, and anti-senescence activity of ceria NPs were explored in vitro, with no significant effects on the coagulation process, hemolysis, or platelet aggregation. In human endothelial cells, ceria did not affect cell viability, although it reduced oxidative stress and inhibited the expression of an inflammatory phenotype. Notably, it reduced telomere shortening, thus demonstrating the potential to counteract premature senescence [240]. However, ceria also impaired neuronal differentiation of neural stem cells [241]. Finally, ceria NPs’ interaction with lipid bilayers has been investigated as concerns were raised for possible cell membrane disruption [242].

Overall, it appears that no major toxicity of nanosized ceria was manifest, albeit some evidence was provided on adverse repercussions on specific biological studies, depending on several parameters, also including the route of administration, the dose, the extent, and frequency of exposure, and so on. There are many parameters that can influence ceria nanoparticle biocompatibility including size, surface charge, and crystalline phase [243]. Therefore, more studies in this area are imperative to make progress in laying the roadmap of nanosized ceria used in biological organisms and in the environment. A key aspect will be to employ models and dosages that are relevant to realistic conditions of exposure depending on the intended use for this nanomaterial. 

## 5. Conclusions

In conclusion, the remarkable redox properties and oxygen-binding ability of ceria have been long-exploited by industry, especially relevant to catalytic applications, preservation, and remediation of the environment and the energy sector. In medicine, nanosized ceria has been attracting growing interest in recent years [244], and we have witnessed the development of a wide number of applications, especially in proof-of-concept studies that used its nanozyme activity either in sensing or to mitigate the effects of oxidative stress associated with many existing pathologies, ranging from cancer to inflammation diseases. In particular, a recent area of investigation that warrants scope for further studies is the application of ceria anti-inflammatory activity to address unmet clinical needs, such as systemic inflammatory syndromes [245] or brain diseases [246]. However, such challenging applications will certainly need many more years of preclinical studies to reach patients [247], as opposed to nanoceria use in dental nanocomposites, where the first clinical trials have already appeared, showing excellent promise [248]. 

The use of greener production methods for nanosized ceria has also been gaining momentum, as well as for other types of nanomaterials [249,250], and the use of biotemplates is becoming one of the most popular approaches. However, thus far, the vast majority of studies either exploited naturally occurring microstructures to impart specific morphology to ceria at the microscale, or natural extracts typically displaying a rather complex mixture of molecular compounds, not often very well defined.

It thus appears that there are unexplored routes for the nanoscale definition of ceria using well-defined biomolecular templates, for instance vastly explored for metal NPs using self-assembling peptides [251]. In line with this principle, it could be possible to use different supramolecular geometries to template ceria into various nanomorphologies, for enhanced properties. For instance, ceria nanorods were recently described with persistent porosity for engineered catalytic sites [252], and nanoengineering methods for the crystal microenvironment are highly sought after to tailor the redox performance of nanosized ceria [253].

Furthermore, using molecular gelators as templates [254] can offer further advantages as it can yield nanostructured gels as functional materials with increased NP stability [255,256]. The combination of different molecular and supramolecular components can lead to a qualitative leap in innovation in areas spanning from pollutants’ removal to catalysis, soft robotics, medicine, and agriculture [257,258,259,260,261]. Therefore, the future for nanosized ceria is bright, especially if combined with other innovative nanomaterials and green approaches.

## Figures and Tables

**Table 1:** Biomolecular templates used for ceria nanostructure definition.

Biotemplate Class	Biomolecule	Ceria Nanomorphology	Crystallite Size (nm)	Ceria NP Size (nm)	Application	Reference
Carbohydrates	Alginate	Spherical	3–5	40–200	Antioxidant	[31]
	Cellulose	Nanoparticles	8	7–10	Catalysis	[32]
	Chitosan	Spherical	8	24	Bioimaging	[33]
	Cyclodextrin	Nanoparticles	n.a.	61	Antioxidant	[34]
	Starch	Irregular	7–8	7–13	Catalysis	[35]
Catechols	PDA ^1^ NPs	Spherical	10	180	Catalysis	[36]
	rGO@PDA ^1,2,3^	Nanosheets	n.a.	3–4	Biosensing	[37]
	Gallate	Nanoflowers	8–13	n.a.	Detection	[38]
Carboxylic acids	Citric acid	Nanocrystals	11–35	n.a.	Catalysis	[39]
Phosphates	DNA	Nanocrystals	5 ± 1	5 ± 1	Antioxidant	[40]
	DNA	Nanocrystals	6 ± 2	6–18 nm	Optoelectronics	[41]
	DNA	Nanocrystals	n.a.	50–400	Catalysis	[42]
	Phytic acid	Nanosheets	n.a.	n.a.	Flame retardant	[43]
Proteins	Albumin	Nanoparticles	n.a.	15	Antioxidant	[44]
	Albumin	Spherical, Nanochains	2	2–100	Catalysis	[45]
	Apoferritin	Nanocrystals	5.0 ± 0.7	5.0 ± 0.7	Catalysis	[46]
	Ferritin	Spherical	n.a.	7	Catalysis	[47]
	Silicatein	Nanocrystals	<3	2.56 ± 0.38	Catalysis	[48]

^1^ PDA = polydopamine. ^2^ rGO = reduced graphene oxide. ^3^ @ denotes core@shell structure.
